# Estimation of the Fraction of Cancer Cells in a Tumor DNA Sample Using DNA Methylation

**DOI:** 10.1371/journal.pone.0082302

**Published:** 2013-12-02

**Authors:** Takamasa Takahashi, Yasunori Matsuda, Satoshi Yamashita, Naoko Hattori, Ryoji Kushima, Yi-Chia Lee, Hiroyasu Igaki, Yuji Tachimori, Masato Nagino, Toshikazu Ushijima

**Affiliations:** 1 Division of Epigenomics, National Cancer Center Research Institute, Tokyo, Japan; 2 Department of Gastroenterological Surgery, Graduate School of Medicine, Osaka City University, Osaka, Japan; 3 Pathology and Clinical Laboratory Division, National Cancer Center Hospital, Tokyo, Japan; 4 Department of Internal Medicine, College of Medicine, National Taiwan University, Taipei, Taiwan; 5 Esophageal Surgery Division, National Cancer Center Hospital, Tokyo, Japan; 6 Division of Surgical Oncology, Department of Surgery, Nagoya University Graduate School of Medicine, Nagoya, Japan; The Chinese University of Hong Kong, Hong Kong

## Abstract

Contamination of normal cells is almost always present in tumor samples and affects their molecular analyses. DNA methylation, a stable epigenetic modification, is cell type-dependent, and different between cancer and normal cells. Here, we aimed to demonstrate that DNA methylation can be used to estimate the fraction of cancer cells in a tumor DNA sample, using esophageal squamous cell carcinoma (ESCC) as an example. First, by an Infinium HumanMethylation450 BeadChip array, we isolated three genomic regions (*TFAP2B*, *ARHGEF4*, and *RAPGEFL1*) i) highly methylated in four ESCC cell lines, ii) hardly methylated in a pooled sample of non-cancerous mucosae, a pooled sample of normal esophageal mucosae, and peripheral leukocytes, and iii) frequently methylated in 28 ESCCs (*TFAP2B*, 24/28; *ARHGEF4*, 20/28; and *RAPGEFL1*, 19/28). Second, using eight pairs of cancer and non-cancer cell samples prepared by laser capture microdissection, we confirmed that at least one of the three regions was almost completely methylated in ESCC cells, and all the three regions were almost completely unmethylated in non-cancer cells. We also confirmed that DNA copy number alterations of the three regions in 15 ESCC samples were rare, and did not affect the estimation of the fraction of cancer cells. Then, the fraction of cancer cells in a tumor DNA sample was defined as the highest methylation level of the three regions, and we confirmed a high correlation between the fraction assessed by the DNA methylation fraction marker and the fraction assessed by a pathologist (r=0.85; p<0.001). Finally, we observed that, by correction of the cancer cell content, CpG islands in promoter regions of tumor-suppressor genes were almost completely methylated. These results demonstrate that DNA methylation can be used to estimate the fraction of cancer cells in a tumor DNA sample.

## Introduction

Contamination of normal cells, such as normal epithelial cells, fibroblasts, and peripheral leukocytes, is almost always present in tumor samples. Such contamination influences the results of cancer genome analyses [1-4], and RNA expression analysis [5,6]. If the fraction of cancer cells in a tumor DNA sample can be readily assessed, we can conduct more accurate analysis by excluding samples with extremely low tumor cell content, and by normalizing the raw data based upon the fraction of cancer cells. Traditionally, a fraction of cancer cells in a tumor sample has been assessed by pathological analysis using neighboring sections. However, preparation of such sections is sometimes difficult or impossible due to sample availability, and, above all, expert pathologists are necessary for such analysis. 

To overcome these issues, technologies to estimate a fraction of cancer cells were recently developed using single nucleotide polymorphism (SNP) microarray and next generation sequencing (NGS) [7-10]. With SNP microarray, the fraction of cancer cells can be calculated by detecting genomic regions with copy number alterations and measuring the degree of allelic imbalance using SNPs in the genomic regions [7-9]. With NGS, tumor-specific mutations can be identified, and the fraction of cancer cells can be calculated based upon the mutant allele frequency [10]. Since these technologies were originally developed for the analysis of SNPs or mutations, they suffer from complicated calculation formulae, expensive reagents, and the necessity of analysing paired normal samples. 

**Figure 1:**
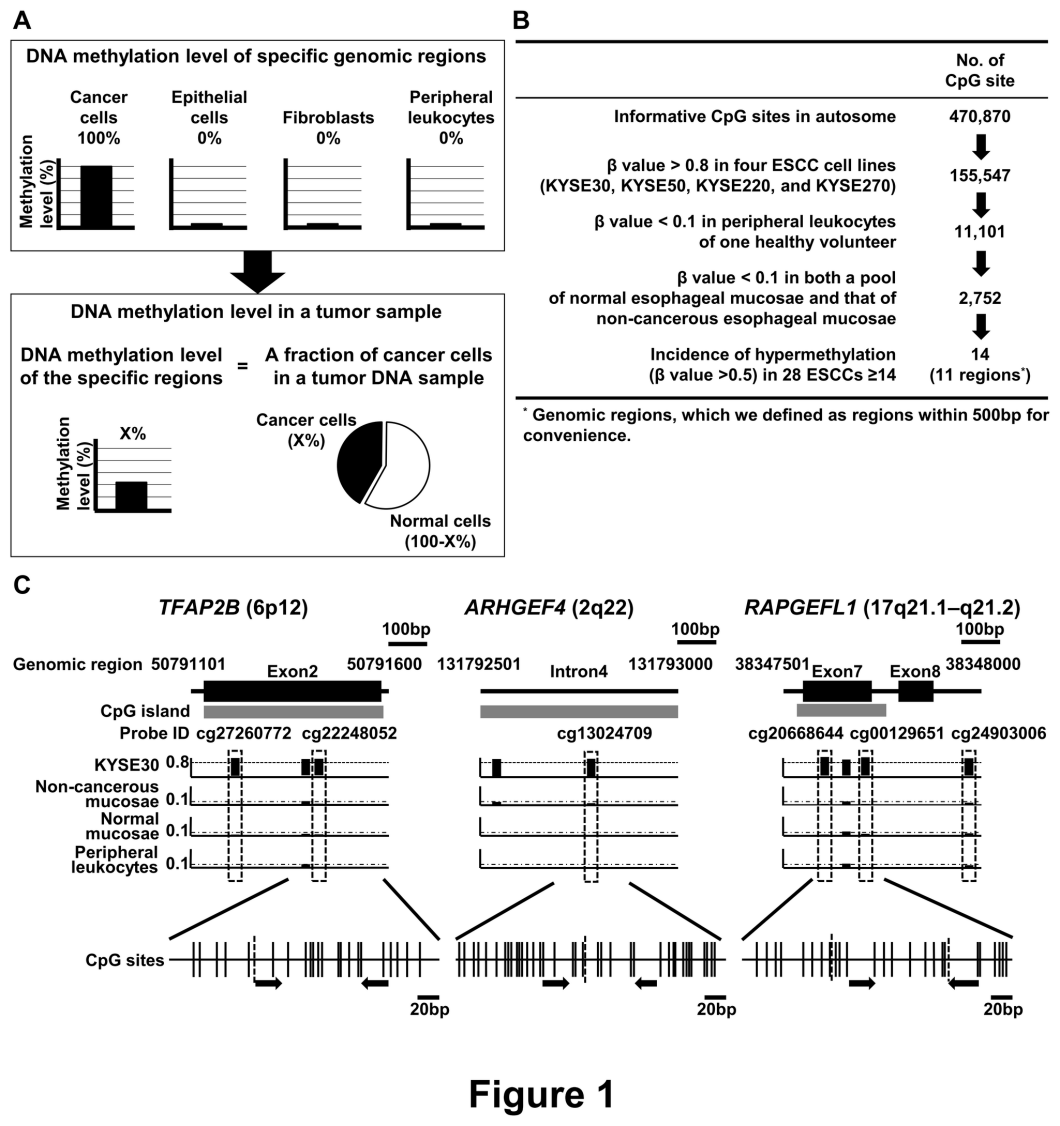
Selection processes of specific genomic regions. (A) The concept of estimation of the fraction of cancer cells in a tumor DNA sample using DNA methylation. (B) Selection of specific genomic regions, which were completely methylated in ESCC cells and completely unmethylated in various normal cells, by genome-wide methylation analysis using an Infinium HumanMethylation450 BeadChip array. Fourteen CpG sites were finally selected, and those were derived from 11 genomic regions. (C) Three genomic regions (TFAP2B, *ARHGEF4*, and *RAPGEFL1*) selected as components of a fraction marker. Gene structure and the location of a CpG island are shown at the top, and the β values of the CpG sites analyzed by the Infinium bead array are shown. The CpG site identified by the Infinium bead array is boxed by a rectangle with a dotted line. A CpG map around the CpG site(s) identified is shown at the bottom. Vertical lines (solid and broken) show CpG sites and broken lines show CpG sites whose β values were measured by the bead array. Closed arrows show primers for MS-HRMA.

DNA methylation is a stable epigenetic modification, and some genomic regions are methylated in a cell type-dependent manner [11-13]. Between cancer and normal cells, many genomic regions are reported to be differentially methylated in many types of cancers, and some of them are causally involved in cancer development and progression [14-18]. Among such differentially methylated genomic regions, some specific genomic regions may be completely methylated in cancer cells but yet completely unmethylated in normal cells, such as normal epithelial cells, fibroblasts, and peripheral leukocytes. If so, DNA methylation levels of the specific genomic regions can reflect the fraction of cancer cells in a sample (Figure 1A), and such estimation of the fraction of cancer cells using DNA methylation can become a useful method for cancer studies. 

In this study, we aimed to demonstrate that DNA methylation could be used as a fraction marker for the estimation of a fraction of cancer cells in a tumor DNA sample, using esophageal squamous cell carcinoma (ESCC) samples as an example. To this end, we conducted a genome-wide methylation analysis to search specific genomic regions completely methylated in ESCC cells and completely unmethylated in various normal cells. 

## Materials and Methods

### Ethics Statement

This study was conducted with the approval of the Institutional Review Board of the National Cancer Center Hospital, the Osaka City University Hospital, and the National Taiwan University Hospital. Written informed consents were obtained from all individuals. 

### Samples and ESCC Cell Lines

A total of 160 ESCC samples were collected at the National Cancer Center Hospital, the Osaka City University Hospital, and the National Taiwan University Hospital. The 160 ESCC samples consisted of 39 biopsy samples and 121 surgical specimens. The biopsy samples were derived from 39 patients who underwent endoscopic biopsy and were diagnosed to have an ESCC (33 male and 6 female; average age = 63, range = 30–78). Samples were stored in RNAlater (Applied Biosystems, Foster City, CA, USA) at -80 °C. The surgical specimens were derived from 121 patients who were diagnosed to have a histologically invasive ESCC and had undergone esophagectomy (98 male and 23 female; average age = 65, range = 41–82). Among the 121 surgical specimens, 25 specimens were obtained from samples embedded in paraffin wax block after fixation with formalin or 70% ethanol, and the other 96 specimens were obtained from samples stored in RNAlater (Applied Biosystems) after resection. Additionally, laser capture microdissection (LCM) was performed for eight surgical specimens embedded in paraffin wax block using LM200 (Arcturus, Mount View, CA, USA) as described [19]. 

Four ESCC cell lines KYSE30, KYSE50, KYSE220, and KYSE270 [20] were purchased from Health Science Research Resources Bank (Osaka, Japan). Genomic DNA was extracted by using the phenol/chloroform method. 

### Genome-wide DNA Methylation Analysis

Genome-wide screening of differentially methylated CpG sites was performed using an Infinium HumanMethylation450 BeadChip array, which covered 482,421 CpG sites (Illumina, San Diego, CA, USA) as described previously [21]. 11,551 CpG sites on the sex chromosomes were excluded, and the remaining 470,870 CpG sites were used for the analysis. The methylation level of each CpG site was represented by a β value, which ranged from 0 (completely unmethylated) to 1 (completely methylated). 

### Methylation-sensitive High Resolution Melting Analysis (MS-HRMA)

For MS-HRMA, first, 1 μg of DNA digested with *Bam*HI was treated with sodium bisulfite and suspended in 40 μL of TE buffer as described [22]. Second, PCR was conducted using 1 μL aliquot of the sodium bisulfite-treated DNA, primers capable of amplifying both methylated and unmethylated DNA (Table S1) [23], SYBR Green I (BioWhittaker Molecular Applications, Rockland, MD, USA), and an iCycler Thermal Cycler (Bio-Rad Laboratories, CA, USA). Third, HRMA of the PCR product was conducted to obtain the melting profile of the PCR product by plotting the negative derivative of fluorescence over temperature (-dF/d*T* vs. *T*), using the iCycler Thermal Cycler software (Bio-Rad Laboratories, Ver. 2.1). Finally, the methylation level of a sample was assessed by comparison of its melting profile with those of fully methylated and unmethylated controls, along with mixtures of 0, 20, 40, 60, 80, and 100% methylated controls. As a fully methylated control, genomic DNA treated with *Sss*I methylase (New England Biolabs, Beverly, MA, USA) was used. As a fully unmethylated control, sodium bisulfite-modified DNAs obtained from non-cancerous esophageal mucosae without methylation of the analyzed regions was used. 

### Genomic DNA Copy Number Analysis

Copy number alterations of the genomic regions were analyzed by quantitative real-time PCR using an iCycler Thermal Cycler (Bio-Rad Laboratories) with SYBR green I (BioWhittaker Molecular Applications). The number of DNA molecules in a sample was measured for three regions flanking the candidate genes and control genes [*ALB* (4q13.3), *GAPDH* (12p13) and *KCNA1* (12p13.32)] located on chromosomal regions with infrequent copy number alterations. The primer sequences are shown in Table S2. The number of DNA molecules of the three candidate genes was normalized to those of the control genes. The normalized number of DNA molecules in a sample was compared with that in human leukocyte DNA to obtain copy number alterations. All the analysis was conducted in triplicate. Significant copy number alterations (gain or loss) were defined as more than a 1.5-fold increase or less than a 0.67-fold decrease. 

### Pathological Analysis of Fraction of Cancer Cells

From paraffin-embedded surgical specimens, serial slice sections with 3-μm thickness were prepared. One section was stained with hematoxylin-eosin, and the remaining sections were used for DNA extraction. An experienced pathologist (R. K.) estimated the fraction of cancer cells by microscopic examination. 

### Statistical Analyses

The correlation between the fraction of cancer cells estimated by the fraction marker and that assessed by the pathologist was analyzed using Pearson's product-moment correlation coefficients. A difference in the mean fractions of cancer cells was analyzed by Student's t test, and the difference in the variances of the cancer cell fraction was analyzed by Levene's test. All the analyses were performed using PASW statistics version 18.0 (SPSS Japan Inc., Tokyo, Japan), and the results were considered significant when p values <0.05 were obtained by a two-sided test. 

## Results

### Selection of Specific Regions by Genome-wide Screening

Genome-wide methylation analysis was performed using DNA of i) 28 ESCCs obtained by endoscopic biopsy, ii) four ESCC cell lines (KYSE30, KYSE50, KYSE220, and KYSE270), iii) peripheral leukocytes of one healthy volunteer, iv) a pool of normal esophageal mucosae of four healthy volunteers, and v) a pool of non-cancerous esophageal mucosae of eight ESCC patients. We searched for CpG sites that were highly methylated (β value > 0.8) in all the four ESCC cell lines and hardly methylated (β value < 0.1) in peripheral leukocytes, the pool of normal mucosae, and the pool of non-cancerous mucosae, and isolated 2,752 CpG sites from 470,870 informative CpG sites. From the 2,752 CpG sites, we selected 14 CpG sites in which the incidence of hypermethylation (β value > 0.5) in the 28 ESCCs was more than 50% (Figure 1B). 

The 14 CpG sites were derived from 11 genomic regions, which we defined as regions within 500bp for convenience (Table 1). From the 11 genomic regions, we excluded *SOX2OT*, which was reported to be highly amplified in ESCC cells [24], and three regions without known genes. For the seven remaining regions, we attempted to design primers for MS-HRMA, which is known as a sensitive and specific method to assess DNA methylation levels [25]. We were able to design primers for three regions (*TFAP2B*, *ARHGEF4*, and *RAPGEFL1*) (Figure 1C). 

**Table 1:** Genomic regions identified by beadchip analysis.

**No**.	**Gene symbol**	**Gene name**	**Chr**	**nt, number**	**Probe ID**	**Relation to CpG island**	**Position to gene**	**Incidence^*a*^**
1	TFAP2B**	Transcription factor AP-2 beta	6	50791202	cg27260772	Island	Body	24
				50791419	cg22248052	Island	Body	14
2	*SOX2OT*	SOX2 overlapping transcript	3	181438216	cg05513806	S_Shore	Body	22
3	ARHGEF4**	Rho guanine nucleotide exchange factor (GEF) 4	2	131792772	cg13024709	Island	Body	20
4	RAPGEFL1**	Rap guanine nucleotide exchange factor (GEF)-like 1	17	38347603	cg20668644	Island	Body	19
				38347968	cg24903006	S_Shore	Body	19
				38347710	cg00129651	Island	Body	14
5	GRK7*	G protein-coupled receptor kinase 7	3	141516705	cg25640519	S_Shore	Body	18
6	*-*		2	200327334	cg07835424	Island	-	18
7	*-*		2	119607885	cg09385093	Island	-	18
8	KCNA3*	Potassium voltage-gated channel, shaker-related subfamily, member 3	1	111217194	cg20302133	Island	1stExon	15
9	BCAT1*	Branched chain amino-acid transaminase 1, cytosolic	12	25055967	cg20399616	Island	Body	14
10	KLF16*	Kruppel-like factor 16	19	1857004	cg04998634	Island	Body	14
11	*-*		1	170630558	cg23089825	Island	-	14

NOTE: **^*a*^**Incidence of frequency of hypermethylation (βvalue > 0.5) in 28 ESCCs. **Primers for HRMA were successfully designed. *Primers for HRMA could not be designed.

### Qualification of the Three Regions as a Fraction Marker

**Figure 2:**
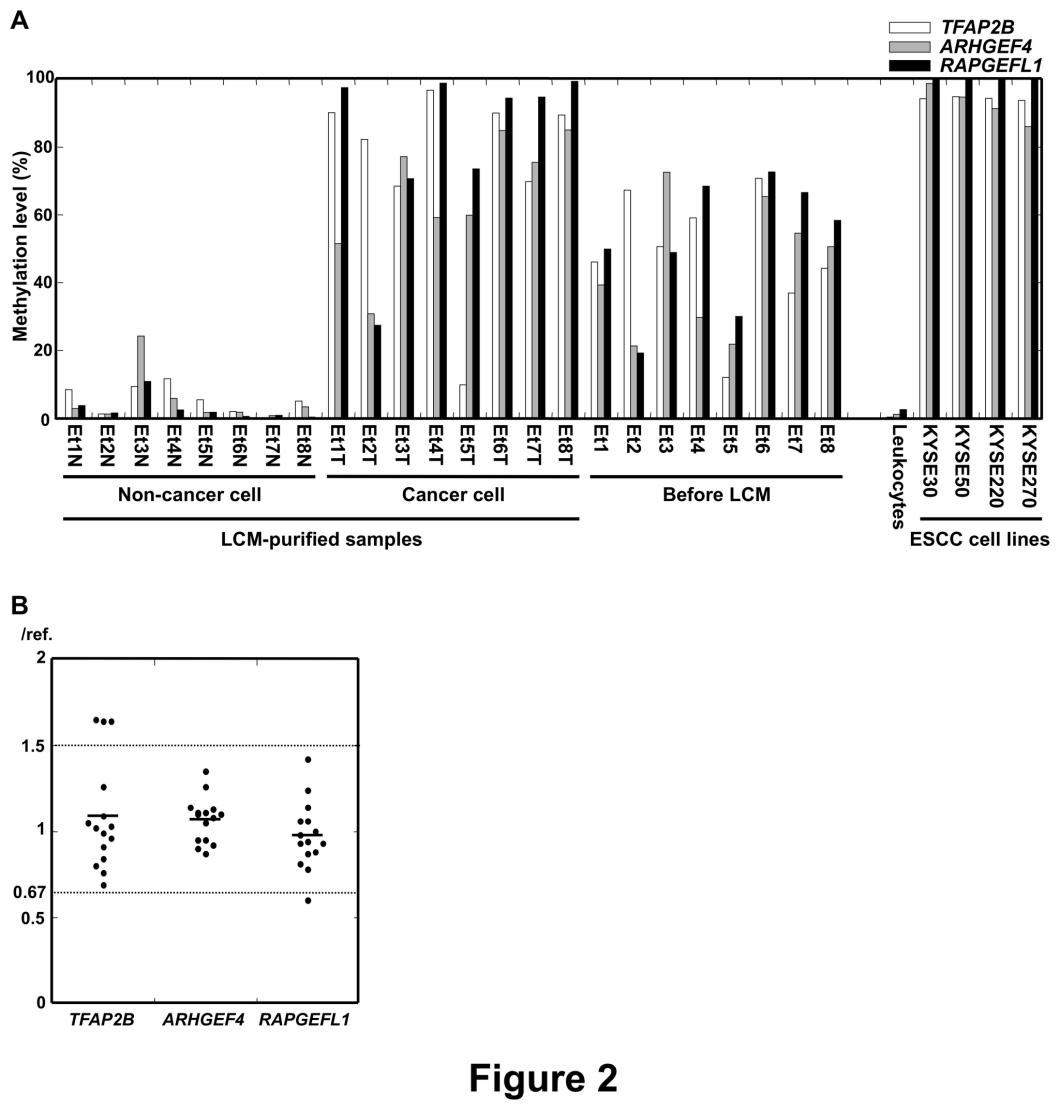
Qualification of the three regions as a fraction marker. (A) Methylation levels of the three regions were analyzed in i) eight LCM-purified non-cancer cell samples from three ESCCs, ii) eight LCM-purified cancer cell samples from the three ESCCs, iii) the eight ESCCs before LCM, iv) peripheral leukocytes of one healthy volunteer, and v) four ESCC cell lines. One or more of the three regions were almost completely methylated in purified cancer cells and cancer cell lines, and all were hardly methylated in non-cancer cells. (B) Copy number alterations of the three regions were analyzed by quantitative PCR using 15 ESCCs.

To confirm that the three regions were completely unmethylated in non-cancerous cells, and completely methylated in cancer cells, we analyzed their methylation levels in i) eight LCM-purified non-cancer cell samples from eight ESCCs, ii) eight LCM-purified cancer cell samples from the eight ESCCs, iii) the eight ESCCs before LCM, iv) peripheral leukocytes of one healthy volunteer, and v) the four ESCC cell lines (Figure 2A). 

In the eight LCM-purified non-cancer cell samples and the peripheral leukocytes, all three regions were almost completely unmethylated, and in the four ESCC cell lines, all of them were completely methylated. In the eight LCM-purified cancer cell samples, at least one of the three regions was almost completely methylated. However, *TFAP2B* in sample Et5T, *ARHGEF4* in Et1T, Et2T, Et4T, and Et5T, and *RAPGEFL1* in Et2T were not completely methylated, possibly due to heterogeneity among cancer cells. Such regions were not suitable as a fraction marker for some specific samples. At the same time, the region with the highest methylation level in the LCM-purified cancer cell samples also showed the highest methylation level in the ESCCs before LCM. Therefore, the highest methylation level of the three regions was considered to reflect the fraction of cancer cells in the ESCCs. 

The methylation level of a region in a sample can be affected by a copy number alteration of the region. Therefore, we analyzed copy number alterations of the three regions using 15 ESCCs (Figure 2B). For *ARHGEF4*, no significant copy number alterations were observed. In contrast, for *TFAP2B* and *RAPGEFL1*, significant copy number alterations were observed at low frequencies (*TFAP2B*, 1.64-1.65 fold changes in three of the 15 ESCCs; *RAPGEFL1*, 0.60-fold change in one of the 15 ESCCs). Assuming that 2-fold or 0.5-fold copy number alterations were present in cancer cells, a deviation of the measured methylation level from the true fraction of cancer cells was calculated to be less than 17.2% (Figure S1). Also, the incidence of significant copy number alterations of *TFAP2B* and *RAPGEFL1* was low (*TFAP2B*, 20%; *RAPGEFL1*, 6.7%). Therefore, the effect of the copy number alterations of *TFAP2B*, and *RAPGEFL1* was considered to be minimal in the estimation of a fraction of cancer cells. Finally, we defined the fraction of cancer cells as the highest methylation level of the three regions. 

### Analysis of the Accuracy of the Fraction Marker

**Figure 3:**
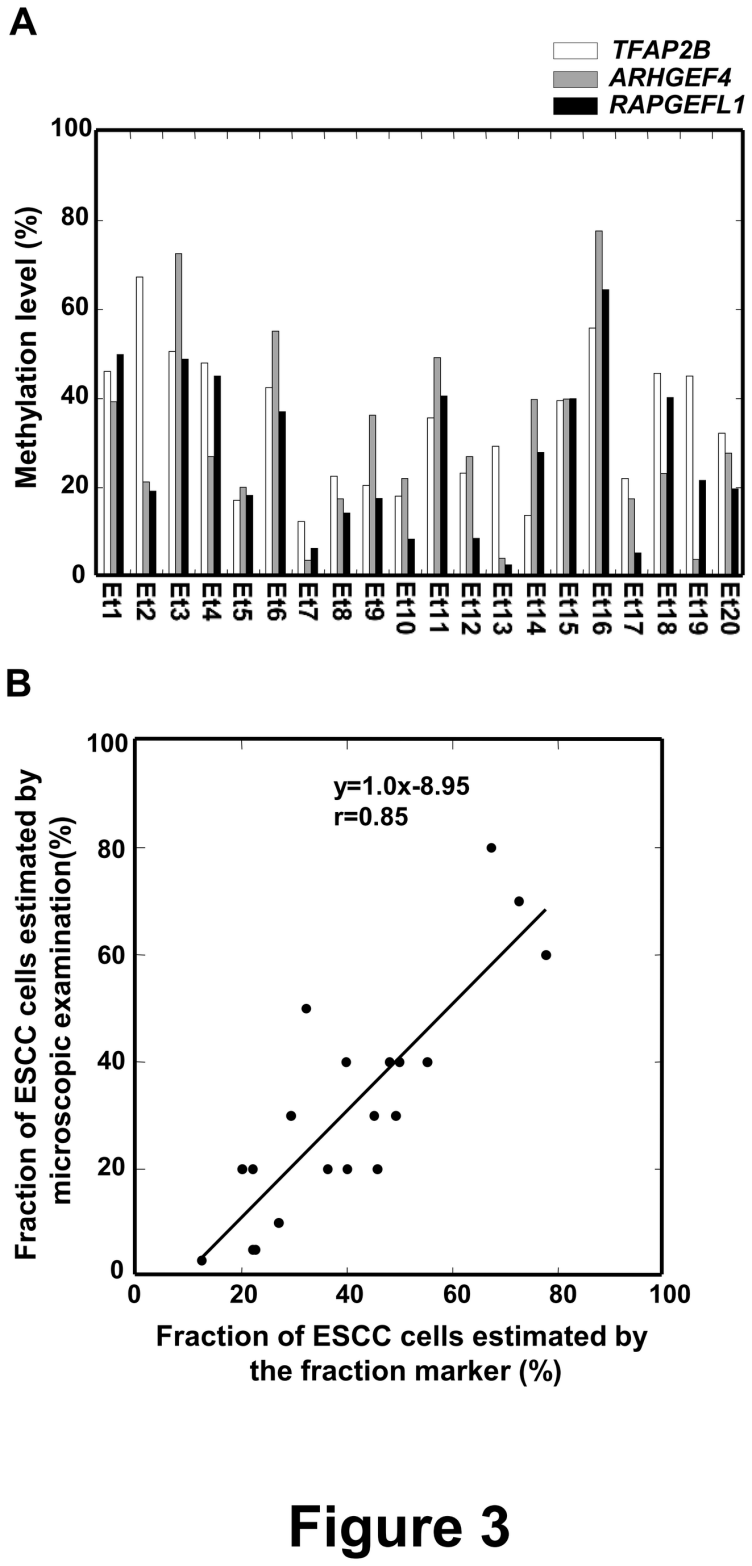
Analysis of the accuracy of the fraction marker. (A) Methylation levels of the three regions were analyzed in 20 ESCCs by MS-HRMA. The fraction of cancer cells in each sample was defined as the highest methylation level of the three regions. (B) Correlation between the fraction of cancer cells estimated by the fraction marker and that assessed by microscopic examination. The correlation between the two methods, calculated using Pearson's product-moment correlation coefficient, was sufficiently high (r=0.85).

We then analyzed whether the cancer cell fraction estimated by the fraction marker really reflected the fraction of cancer cells estimated by microscopic examination. We measured methylation levels of the three regions in 20 ESCCs (Figure 3A), and estimated the fraction of cancer cells in each sample, using the highest value of the three regions. In the 20 ESCCs, *TFAP2B*, *ARHGEF4*, and *RAPGEFL1* showed the highest values in nine, nine, and two samples, respectively. Independently, the fraction of cancer cells was estimated by microscopic examination of serial sections. We were able to observe a good correlation between the two methods (r=0.85; p<0.001) (Figure 3B). This result confirmed that the fraction of cancer cells estimated by the fraction marker accurately reflected the true fraction of cancer cells in a tumor sample. 

### Application of the Fraction Marker

**Figure 4:**
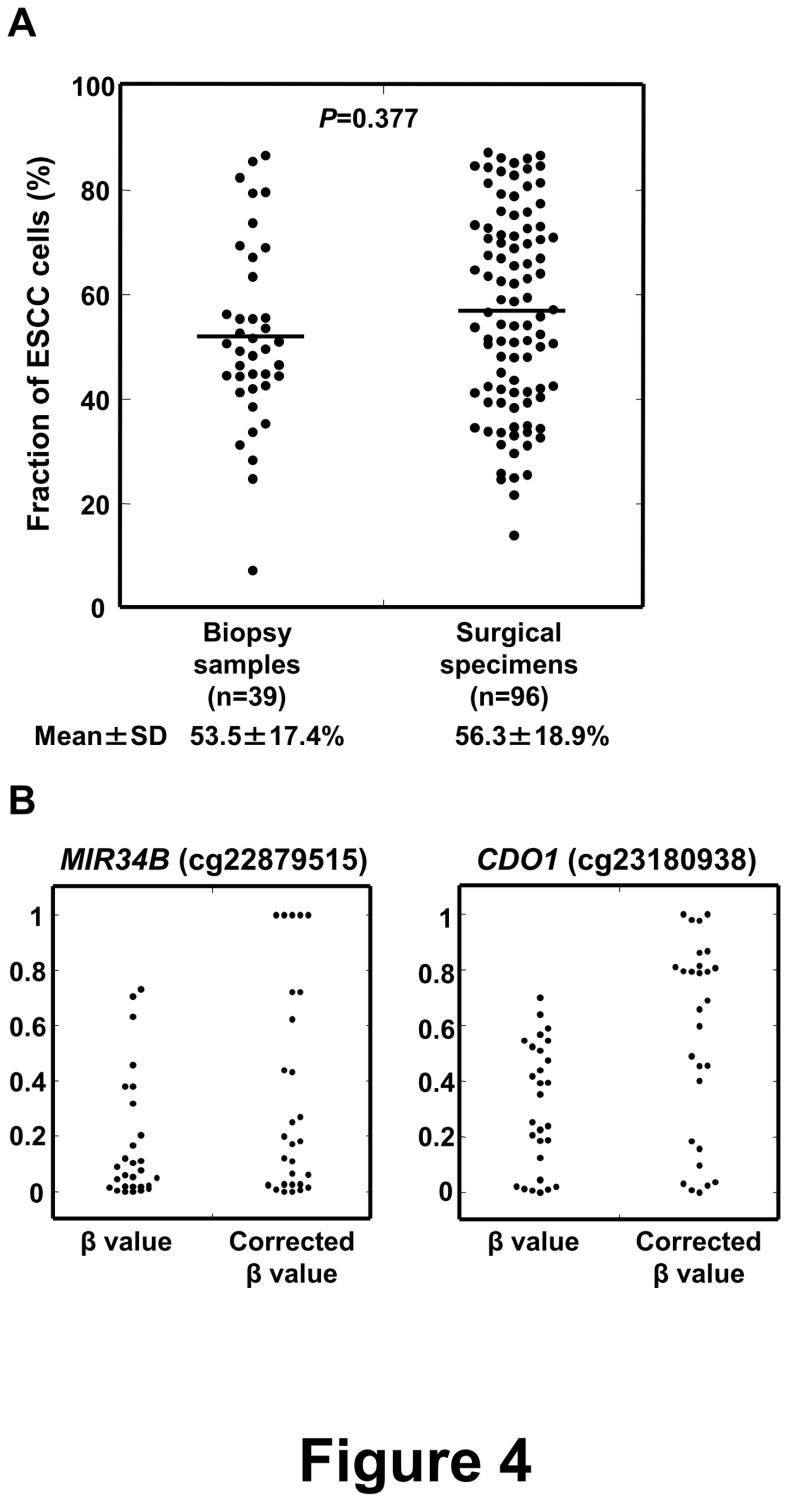
Application of the fraction marker. (A) Application of the fraction marker to comparison of the fraction of cancer cells between biopsy samples and surgical specimens. Fractions of cancer cells in 39 biopsy samples and 96 surgical specimens were estimated by the fraction marker, and no significant difference was observed. (B) Application of the fraction marker to DNA methylation analysis. Raw β values of two CpG sites [cg22879515 (MIR34B), cg23180938 (CDO1)] were corrected by the cancer cell content in 28 ESCCs. Some ESCCs had almost complete methylation (β value ≥ 0.8).

We applied the fraction marker to compare the fractions of cancer cells between biopsy samples and surgical specimens. The fractions of cancer cells in 39 biopsy samples and 96 surgical specimens were assessed using the fraction marker. Among the 135 samples, *TFAP2B*, *ARHGEF4*, and *RAPGEFL1* had the highest values in 60, 37, and 38 samples, respectively, and the highest values were defined as the fractions of cancer cells in the samples. The mean fraction of cancer cells in the biopsy samples (mean±SD, 53.5±17.1%; range, 7.3-86.7%) was not significantly different from that in the surgical specimens (56.7±18.9%; 14.0-87.3%) (p=0.377) (Figure 4A). The variances were large in both the biopsy samples and in the surgical specimens, and there was no significant difference between the biopsy samples and surgical specimens (p=0.076). This confirmed that contamination of normal cells must be taken care of for analysis of tumor DNA samples with unknown cancer cell contents. 

The fraction marker was also applied to exclude the effect of contamination of normal cells in DNA methylation analysis. For this purpose, we selected CpG sites within 200 bp from transcription start sites (TSS200) for two tumor-suppressor genes (cg22879515 (*MIR34B*) [26], and cg23180938 (*CDO1*) [27]), and assessed distributions of their β values obtained in the initial genome-wide methylation analysis. CpG sites in TSS200 of tumor-suppressor genes were used since they were expected to have no or complete methylation in cancer samples because of the growth advantage conferred by their methylation. The βvalues of the two CpG sites were less than 0.1 in the peripheral leukocytes, the pool of normal mucosae, and the pool of non-cancerous mucosae, and the two CpG sites were considered to be almost completely unmethylated in normal cells. Raw β values of the two CpG sites and βvalues corrected for the cancer cell content [Corrected β value = β value/(a fraction of ESCC cells in the sample (%)/100)] were then compared (Figure 4B). Using the β values corrected by the fraction marker, some samples showed complete methylation (β value ≥ 0.8), some samples showed almost no methylation (β value < 0.2). This result suggested that by using the fraction marker, we were able to minimize the effect of contamination of normal cell DNA in tumor DNA samples for methylation analysis. 

## Discussion

In this study, the fraction of cancer cells in an ESCC sample was successfully estimated using DNA methylation levels as a fraction marker. To achieve this, we isolated three genomic regions (*TFAP2B*, *ARHGEF4*, and *RAPGEFL1*) that were highly and frequently methylated in ESCC cells, but not in normal cells, by genome-wide methylation analysis. This is the first study in which a fraction of cancer cells in a tumor DNA sample was estimated using DNA methylation. Recently, differentially methylated genomic regions between cancer and normal cells have been identified in many types of cancers [14-18]. Therefore, we can expect to identify specific genomic regions highly and frequently methylated in in the other types of cancer cells, but not in normal cells. By measuring DNA methylation levels of such specific genomic regions, the fraction of cancer cells can be also estimated in DNA samples of other types of cancers. 

The accuracy of the fraction marker was verified by comparing the fraction of cancer cells estimated by the fraction marker with that estimated by microscopic examination. A high accuracy was supported by a good correlation between the fractions of cancer cells estimated by the two methods (r=0.85). Also, the methylation levels of two CpG sites in TSS200 of two tumor-suppressor genes (*MIR34B* and *CDO1*) were corrected, and some cancer samples showed almost complete methylation. Scatter plot analysis of the β values before and after the correction showed that some of the samples with low β values had large increases (Figure S2). Practically, our fraction marker has the advantage over microscopic examination because it can be used for samples only with DNA and the dedication of experienced pathologists is not necessary. Our fraction marker also has an advantage over approaches using SNP microarray and NGS because it can be used even without paired normal samples, and can be analyzed relatively easily and inexpensively. Therefore, the use of a fraction marker has reasonable accuracy, and practical advantages over the other methods. 

One limitation of our fraction marker is that the estimation can be influenced by heterogeneity among cancer cells because the three regions are not always completely methylated in cancer cells. Since cancer cells are theoretically clonal, the heterogeneity was considered to be due to methylation or demethylation during cancer progression. To minimize the effect of the heterogeneity among cancer cells, three regions were selected as those with the highest incidence of hypermethylation (βvalue > 0.5) among the 28 ESCCs. We also confirmed that, using eight paired LCM-purified cancer and non-cancer cell samples, at least one of the three regions was almost completely methylated in any cancer cells. Another limitation is that copy number alterations can influence the estimation. To exclude the effects of copy number alterations on the estimation, we also investigated copy number alterations of the three regions in 15 ESCCs, and confirmed that copy number alterations that could cause a large deviation of the estimation of the fraction of cancer cells were not observed in the three regions. Owing to these characteristics, the fraction marker made of the three regions can be used for the vast majority of ESCCs. Finally, in sample Et3N, a LCM-purified non-cancer cell sample, the three regions were slightly methylated. This was considered to be due to the incomplete purification by LCM because boundaries between cancer cell clusters and non-cancerous cell clusters were extremely unclear in this sample. 

In conclusion, DNA methylation can be used for the estimation of the fraction of cancer cells in a tumor DNA sample. The estimation is considered to be a practical and accurate method for molecular analysis of cancer tissues in which normal cells are almost always contaminated. The DNA methylation fraction marker is expected to be highly advantageous in many aspects of cancer research.

## Supporting Information

Figure S1 **Measured methylation level and true fraction of cancer cells.** Assuming a 2-fold copy number gain was present in cancer cells, the true fraction of cancer cells was calculated as the [Measured methylation level (%)/(200-Measured methylation level (%))]x100. Assuming a 0.5-fold copy number loss was present in cancer cells, the true fraction of cancer cells was calculated as the [2xMeasured methylation level (%)/(100+Measured methylation level (%))]x100. A deviation of the measured methylation level from the true fraction of cancer cells was calculated to be less than 17.2% both in 2-fold gain and in 0.5-fold loss.
(TIF)

Figure S2 **Comparison of β values before and after correction.** Raw β values of two CpG sites [cg22879515 (*MIR34B*), cg23180938 (*CDO1*)] were corrected by the cancer cell content in the 28 ESCCs. The X-axis shows the raw β values, and the y-axis shows the β values after the correction.
(TIF)

Table S1 **Primers and conditions for MS-HRMA.**
(DOCX)

Table S2 **Primers and conditions for quantitative PCR.**
(DOCX)
