# Supplementary figures and images for: Estimation of the Fraction of Cancer Cells in a Tumor DNA Sample Using DNA Methylation
Source: PLoS One. 2013 Dec 2;8(12):e82302. doi: 10.1371/journal.pone.0082302 (PMC3846724; doi:10.1371/journal.pone.0082302)

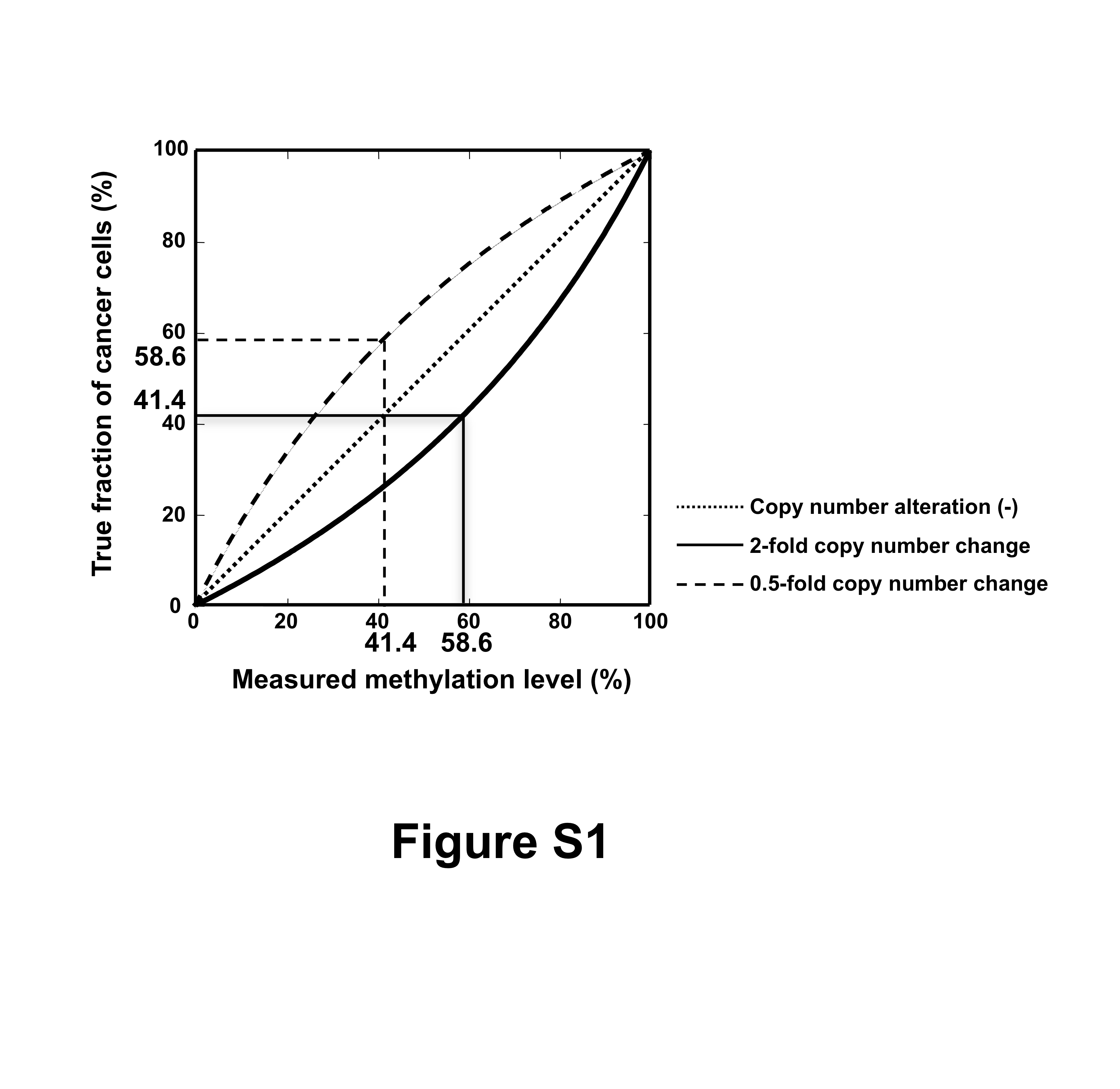

Supplement: Figure S1 — Measured methylation level and true fraction of cancer cells. Assuming a 2-fold copy number gain was present in cancer cells, the true fraction of cancer cells was calculated as the [Measured methylation level (%)/(200-Measured methylation level (%))]x100. Assuming a 0.5-fold copy number loss was present in cancer cells, the true fraction of cancer cells was calculated as the [2xMeasured methylation level (%)/(100+Measured methylation level (%))]x100. A deviation of the measured methylation level from the true fraction of cancer cells was calculated to be less than 17.2% both in 2-fold gain and in 0.5-fold loss. (TIF) [file pone.0082302.s001.tif]

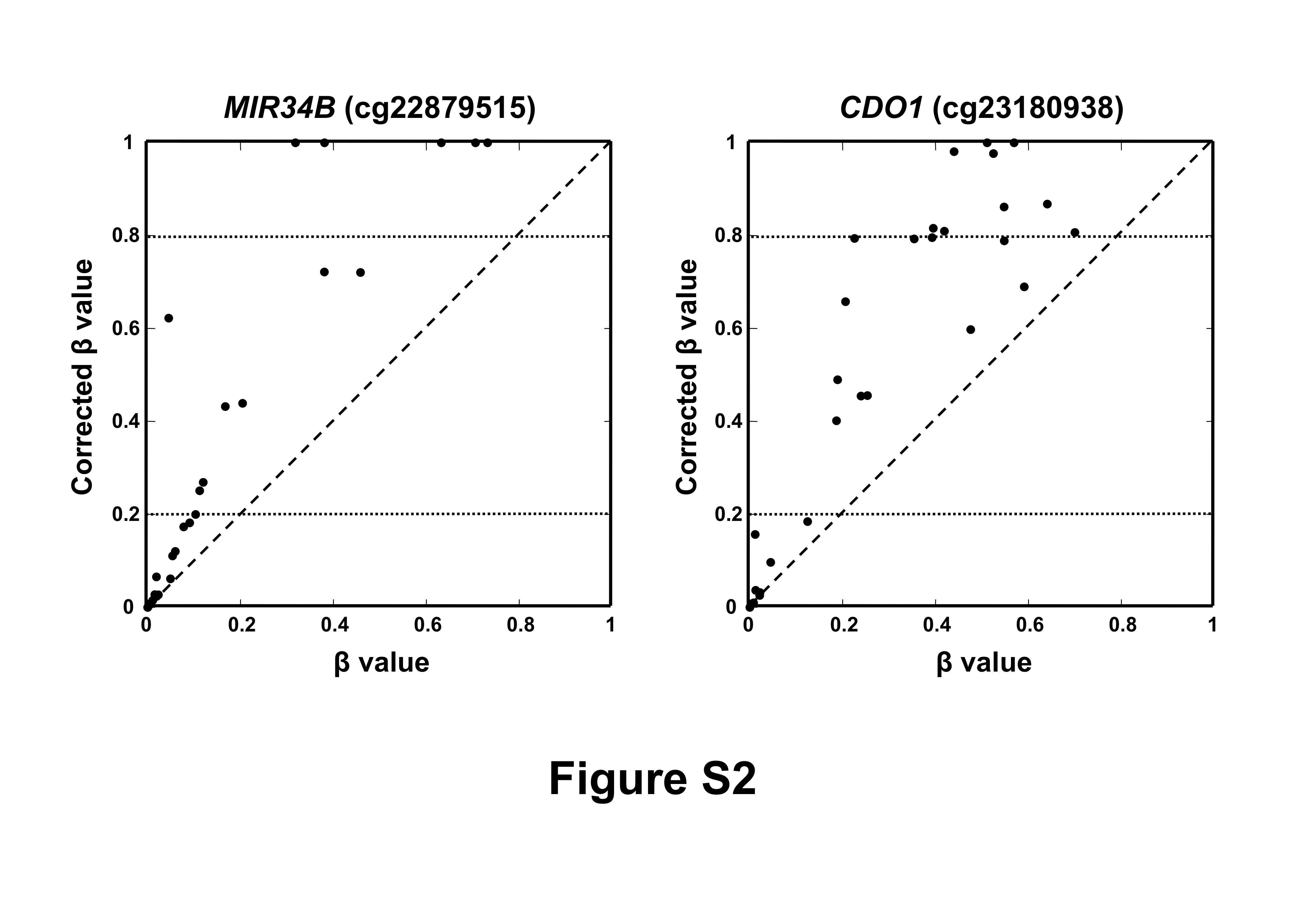

Supplement: Figure S2 — Comparison of β values before and after correction. Raw β values of two CpG sites [cg22879515 (MIR34B), cg23180938 (CDO1)] were corrected by the cancer cell content in the 28 ESCCs. The X-axis shows the raw β values, and the y-axis shows the β values after the correction. (TIF) [file pone.0082302.s002.tif]
