# Supplementary material for: Estimation of the Fraction of Cancer Cells in a Tumor DNA Sample Using DNA Methylation
Source: PLoS One. 2013 Dec 2;8(12):e82302. doi: 10.1371/journal.pone.0082302 (PMC3846724; doi:10.1371/journal.pone.0082302)
Supplement: Table S1 — Primers and conditions for MS-HRMA. (DOCX) [file pone.0082302.s003.docx]

## **Table S1. Primers and conditions for MS-HRMA.**

| **Gene symbol** | **Primer sequence** | | **Length (bp)** | **Annealing temperature (˚C)** |
| --- | --- | --- | --- | --- |
|  | **Forward** | **Reverse** |  |  |
| *RAPGEFL1* | GGTTAATTTGGAGTTGTTGTTGTAG | CTTAATAAACTTCTTAAACAACTAC | 118 | 59 |
| *TFAP2B* | GTTTTGTTTTAGTTTTTGGGTTTTG | CAARAAAAAACTATCACCCATACCC | 123 | 63 |
| *ARHGEF4* | TGTTTTGTTGTGTTGTAATTTGTT | AACCAACAAAAAAATCAATAC | 106 | 57 |
