# Supplementary material for: Estimation of the Fraction of Cancer Cells in a Tumor DNA Sample Using DNA Methylation
Source: PLoS One. 2013 Dec 2;8(12):e82302. doi: 10.1371/journal.pone.0082302 (PMC3846724; doi:10.1371/journal.pone.0082302)
Supplement: Table S2 — Primers and conditions for quantitative PCR. (DOCX) [file pone.0082302.s004.docx]

## **Table S2. Primers and conditions for quantitative PCR.**

| **Gene symbol** | **Chr** | **Genomic region** | **Primer sequence** | | **Length (bp)** | **Annealing temperature (˚C)** |
| --- | --- | --- | --- | --- | --- | --- |
|  |  |  | **Forward** | **Reverse** |  |  |
|  | 17q21.1 | 38346594-38346740 | GGTGAGGAGTTTTTGGATAG | TTGATGCCCAGTGCGGTGAA | 147 | 58 |
| *RAPGEFL1* |  | 38347895-38348037 | GCTGTCAGCCGCCTTCGACT | CCCTCCAACTGGCGCAAAGA | 143 | 66 |
|  |  | 38348396-38348530 | ATCCGAGGCTCCCATGTAAC | GCCAAACCCACTCACCGTCA | 135 | 57 |
|  | 6p12 | 50789982-50790099 | GTCTTCCTTTGAGCGCCTTT | TCCCCTCCCAGCTGACTCAT | 118 | 57 |
| *TFAP2B* |  | 50792215-50792353 | AGAAACTCAGAACGCCATAA | AGTGTTAATATAGGCGGAGG | 139 | 52 |
|  |  | 50792333-50792459 | GCCTCCGCCTATATTAACAC | CTGCAGATGGGATAGTGGGT | 127 | 55 |
|  | 2q22 | 131784295-131784395 | AATGTCTCGTAATGCCAATC | CCTAGGCACACCAAATAGTT | 101 | 54 |
| *ARHGEF4* |  | 131793759-131793898 | AGCGCAATAGCATCGGAGGT | CACTCGGGAGGTACTCTAAT | 140 | 54 |
|  |  | 131794566-131794680 | GCAGCAGCAGCTCATGATCT | GCACAGAGCCCTGGGTTATT | 115 | 59 |
| *ALB* | 4q13.3 | 74274349-74274498 | TCTTCGTGAAACCTATGGTGA | TCATGAAAAGCAGTGCACA | 150 | 60 |
| *GAPDH* | 12p13 | 6641903-6642005 | ATGACAACAGCACACTACGTCAG | TTCCCCTCTGCCTCATGCTT | 103 | 60 |
| *KCNA1* | 12p13.32 | 5020546-5020800 | TGACGGTGATGTCTGGGGAG | GGTTGCGGTCGAAGAAGTAC | 255 | 56 |
